# Epidemiological and molecular characteristics of emergent dengue virus in Yunnan Province near the China-Myanmar-Laos border, 2013–2015

**DOI:** 10.1186/s12879-017-2401-1

**Published:** 2017-05-08

**Authors:** Ting-Song Hu, Hai-Lin Zhang, Yun Feng, Jian-Hua Fan, Tian Tang, Yong-Hua Liu, Liu Zhang, Xiao-Xiong Yin, Gang Chen, Hua-Chang Li, Jin Zu, Hong-Bin Li, Yuan-Yuan Li, Jing Yu, Fu-Qiang Zhang, Quan-Shui Fan

**Affiliations:** 1 Center for Disease Control and Prevention, Chengdu Military Region, 168 Daguan Road, Kunming, 650032 People’s Republic of China; 2 Yunnan Institute of Endemic Diseases Control and Prevention, Dali, Yunnan People’s Republic of China; 3 Xishuangbanna Center for Disease Control and Prevention, Jinghong, Yunnan People’s Republic of China; 4 Ruili Center for Disease Control and Prevention, Ruili, Yunnan People’s Republic of China; 5 Lincang Center for Disease Control and Prevention, Lincang, Yunnan People’s Republic of China; 6 The Postdoctoral Programme of Kunming General Hospital, Chengdu Military Region, Kunming, People’s Republic of China

**Keywords:** Dengue fever, Dengue virus, Molecular epidemiology, Public health, E gene, Homology, Phylogenetic analysis

## Abstract

**Background:**

Yunnan Province is located in southwestern China and neighbors the Southeast Asian countries, all of which are dengue-endemic areas. In 2000–2013, sporadic imported cases of dengue fever (DF) were reported almost annually in Yunnan Province. During 2013–2015, we confirmed that a large-scale indigenous DF outbreak emerged in cities of Yunnan Province near the China-Myanmar-Laos border.

**Methods:**

Epidemiological characteristics of DF in Yunnan Province during 2013–2015 were evaluated by retrospective analysis. A total of 232 dengue virus (DENV)-positive sera were randomly collected for sequence analysis of the capsid/premembrane region of DENV from patients with DF in Yunnan Province. The envelope gene of DENV isolates was also amplified and sequenced. Phylogenetic analyses were performed using the neighbor-joining method with the Tajima-Nei model.

**Results:**

Phylogenetically, all DENV-positive samples could be classified into DENV-1 genotype I and DENV-2 Asian I genotype during 2013–2015 and DENV-4 genotype I in 2015 from Ruili City; and DENV-3 genotype II in 2013 and DENV-2 Cosmopolitan genotype in 2015 from Xishuangbanna Prefecture.

**Conclusions:**

Our results indicated that imported DF from patients from Laos and Myanmar was the primary cause of the DF epidemic in Yunnan Province. Additionally, DENV strains of all four serotypes were identified in indigenous cases in Yunnan Province during the same time period, while the dengue epidemic pattern observed in southwestern Yunnan showed characteristics of a hypoendemic nature: circulation of DENV-1 and DENV-2 over consecutive years.

**Electronic supplementary material:**

The online version of this article (doi:10.1186/s12879-017-2401-1) contains supplementary material, which is available to authorized users.

## Background

Dengue is a mosquito-borne disease caused by the dengue virus (DENV), which is transmitted by *Aedes aegypti* and *Aedes albopictus* mosquitoes that are present in most tropical and sub-tropical regions worldwide [1, 2]. As a member of the family *Flaviviridae* and the genus *Flavivirus*, DENV has four distinct serotypes: DENV-1, DENV-2, DENV-3, and DENV-4 [3]. The disease is prevalent throughout tropical and subtropical regions of the world and is the most rapidly spreading disease, with a 30-fold increase in global incidence over the past 50 years. It is estimated that 390 million dengue infections occur worldwide annually [4, 5]. Complex factors, including climate change, poor vector control, virus evolution, increased population mobility, uncontrolled urbanization, and the lack of effective vaccines, have aggravated the incidence and geographic expansion of dengue [6–8].

Epidemic dengue outbreaks have been reported in Asia and almost all countries in Southeast Asia, including Laos, Myanmar, Indonesia, Cambodia, Malaysia, Philippines, Thailand, and Vietnam [9–18]. Since the first reported dengue outbreak in 1978 in Foshan, Guangdong Province, China, outbreaks have been reported in Hainan, Guangxi, Guangdong, Fujian, Zhejiang, and Yunnan Provinces [19–24]. Moreover, DENV strains of all four serotypes have been identified in southeastern China.

Yunnan Province, which comprises 16 prefectures and 129 counties, is located in Southwest China. It has tropical and subtropical regions and shares a 4060-km border with its neighboring countries Vietnam, Laos, and Myanmar, all of which are dengue-endemic areas. Since 2000, sporadic imported cases of dengue fever (DF) have been reported almost annually in Yunnan Province, China [24]. In 2008, a reported epidemic of DF was imported from Mujie City, Myanmar to Ruili City, Yunnan Province, China [25]. Since 2013, one large-scale dengue outbreak has occurred in Xishuangbanna and Dehong Prefectures of Yunnan Province, which border Laos and Myanmar [23, 26]. From 2013 to 2015, dengue outbreaks have been reported annually in these areas.

In the current study, we analyzed the phylogenetic, molecular, and epidemiological characteristics of DENV outbreaks and their origins in Yunnan Province that occurred from 2013 to 2015.

## Methods

### DF case data sources

The case information in this study was retrieved in a blinded manner from datasets preserved at the Xishuangbanna Center for Disease Control and Prevention (CDC), Lincang CDC, and Dehong CDC. The patients’ acute-phase sera (collected within 1–5 days of illness onset) were also obtained from these CDCs. DF case data were obtained from an infectious diseases database report that was officially compiled by the Yunnan Provincial CDC and Chinese CDC. Detailed information was obtained by epidemiologic investigations. Epidemiological characteristics of DF in Yunnan Province during 2013–2015 were descriptive with a retrospective analysis (including temporal and regional distribution, seasonal distribution of dengue cases, demographic distribution, and occupational distribution of DF patients). These data were analyzed by Graphpad Prism v5.0 (GraphPad Software Inc.). Characteristics of age distribution were compared between different age groups using one-way analysis of variance (one-way ANOVA) and Tukey’s multiple comparison test. Results with *P* values of less than 0.05 were considered statistically significant.

### Case definitions and dengue diagnosis

DF was defined according to diagnostic criteria for DF (WS 216–2008) of the Health Department of China. Criteria for clinical-diagnosed DF included a history of mosquito bite and DF symptoms. A laboratory-confirmed case constituted the detection of DENV non-structural protein 1 (NS1) antigen in acute-phase serum. In this study, all DF cases in Xishuangbanna, Dehong, and Lincang Prefectures in 2013–2015 were laboratory-confirmed cases in which DENV NS1 antigen was detected by the Dengue Ag Rapid Test (CTK Biotech, Inc., San Diego, CA, USA).

### Serotype identification and amplification of the capsid/premembrane region (*CprM*) and envelope (*E*) gene

A total of 232 DENV-positive sera were randomly collected to amplify and sequence the *CprM* gene of DENV from patients with DF. Nucleic acids were extracted using an AxyPrep Body Fluid Viral DNA/RNA Miniprep Kit (Axygen, Inc., USA). Reverse transcription PCR (RT-PCR) of viral RNA was performed using the SuperScript® III One-Step RT-PCR System with Platinum®Taq Kit (Invitrogen, Inc., USA). Testing for amplification of the *CprM* gene and serotype identification were performed by RT-PCR after RNA extraction using serotype identification primers as previously described by Lanciotti et al. [27]. The *E* gene was amplified with the following primers: DENV1 EF, 5′CAGATACAAAGAGTGGAGACTTGG3′ and DENV1 ER, 5′ATTTGCTTCCACATGATGTTCTC3′; DENV2 EF, 5′TGGATGTCATCAGAAGGGG3′ and DENV2 ER, 5′GGTGTTATTTGTTTCCACATTAG3′; DENV3 EF, 5′AGCTTGGAGACAAGTCGAGAAG3′ and DENV3 ER, 5′CCACAATAGATTCTCCATTCTGG3′; DENV4 EF, 5′AACACCACATTCAGGAATGGG3′ and DENV4 ER, 5′CAGTGAGGTCATGTCCTCCTTC3′. *CprM* and *E* genes were amplified and cloned into the pMD18T vector (Takara, Inc., China.) for sequencing. DNA was sequenced using the ABI BigDye Terminator Cycle Sequencing protocol on an ABI 3730 sequencer (Applied Biosystems, Foster City, CA, USA).

### DENV isolation

Virus isolation was carried out in *Ae. albopictus* mosquito C6/36 cells, which were purchased from the ATCC. Acute-phase serum positive for DENV NS1 antigen was diluted 1:20 with Dulbecco’s minimum essential medium (DMEM) (Life Technologies, USA) and inoculated into C6/36 cells for 1–2 h. Inoculated cells were maintained in DMEM supplemented with 100,000 U/mL penicillin, 100 μg/mL streptomycin, and 2% fetal bovine serum (Life Technologies) at 28 °C in 5% CO_2_. Infected cells were subcultured 3 times and observed daily for the occurrence of cytopathic effects (CPE). Upon CPE in the third subculture, the supernatant was collected and stored at −80 °C until further analysis.

### Phylogenetic analysis of isolated DENV

Nucleotide sequences were analyzed using DNAman version 6.0 and compared with sequences available from the BLAST database (http://blast.ncbi.nlm.nih.gov/Blast.cgi). Phylogenetic analyses were performed using the neighbor-joining method with the Tajima-Nei model (MEGA, version 6.0; http://www.megasoftware.net/) [28]. Bootstrap values ≥70%, calculated from 1000 replicates, are shown at the tree branches. DENV genotype was analyzed by including related reference sequences with known genotypes in the phylogenetic tree [19, 21–23, 26, 29].

### Ethics statement

All participants were informed of the aims of the study and the procedures involved in study participation at enrollment; written informed consent was obtained before sample collection. The study was approved by the Institutional Ethical Committee of the Center for Disease Control and Prevention, Chengdu Military Region, the People’s Republic of China.

## Results

### Epidemiological characteristics

#### Temporal and regional distribution

**Fig. 1:**
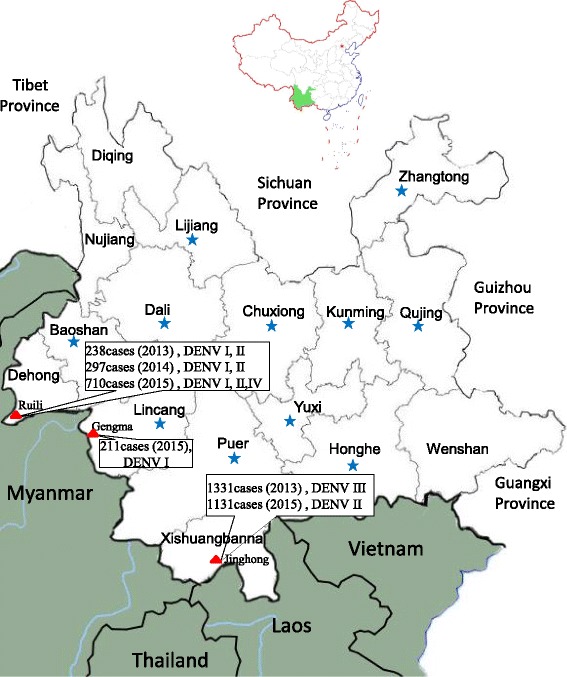
Regional distribution of dengue fever cases in Yunnan Province, China, 2013–2015. The *green* region is Yunnan Province, which comprises 16 prefectures and is located in Southwest China. The cities with large-scale dengue outbreaks are highlighted using *red* triangles (including indigenous and imported cases), and sporadic imported cases of dengue fever evaluated in this paper are highlighted using blue pentagrams. The maps were generated and modified with Photoshop CS 8.0.1

From 2013 to 2015, 4019 dengue cases were reported in 13 prefectures of Yunnan Province (Fig. 1), including 3122 indigenous cases and 897 imported cases. Indigenous dengue outbreaks were reported in Xishuangbanna (2013 and 2015), Dehong (2013–2015), and Lincang (2015) Prefectures, which are located southwest of the province along the Laos and Myanmar borders. In Gengma County (Lincang Prefecture), indigenous dengue infections occurred in rural villages. However, in Jinghong City (Xishuangbanna Prefecture) and Ruili City (Dehong Prefecture), the endemic of indigenous dengue cases mainly occurred in urban areas. Sporadic imported dengue cases were also observed in Kunming, Qujing, Yuxi, Baoshan, Zhaotong, Lijiang, Puer, Chuxiong, Honghe, and Dali Prefectures (Table 1).

**Table 1:** Regional distribution of dengue fever cases in Yunnan Province, China, 2013 to 2015

Prefecture	2013		2014		2015		Total
	Indigenous	Imported	Indigenous	Imported	Indigenous	Imported	
Kunming	0	20	0	16	0	16	52
Qujing	0	0	0	1	0	5	6
Yuxi	0	1	0	0	0	2	3
Baoshan	0	2	0	3	0	6	11
Zhaotong	0	0	0	2	0	2	4
Lijiang	0	1	0	0	0	1	2
Puer	0	1	0	0	0	6	7
Lincang	0	0	0	1	96	104	201
Chuxiong	0	0	0	1	0	0	1
Honghe	0	0	0	0	0	3	3
Wenshan	0	0	0	0	0	0	0
Xishangbanna	1287	44	0	0	1089	42	2462
Dali	0	1	0	2	2	0	5
Dehong	145	93	139	158	309	418	1262
Nujiang	0	0	0	0	0	0	0
Diqing	0	0	0	0	0	0	0
Total							4019

In 2013, the first (emergent) indigenous dengue outbreak occurred in Xishuangbanna; 1331 cases were reported, including 1269 in Jinghong City (1253 indigenous and 16 imported cases), 45 in Mengla County (34 indigenous and 11 imported cases), and 17 imported cases in Menghai County. In 2015, a re-emerging, large-scale indigenous DF outbreak was reported in Xishuangbanna, which was triggered by imported cases (patients) from Laos and Myanmar. A total of 1131 cases were reported, including 1089 indigenous cases and 42 imported cases. Importantly, 1046 of the indigenous cases and 8 of the imported cases were reported in Jinghong City.

Since 2013, epidemics of DF have been reported annually in Dehong Prefecture, with one large-scale outbreak occurring in Ruili City. From 2013 to 2015, a total of 1262 clinically/laboratory-confirmed dengue cases were reported in Ruili City, of which 593 were indigenous and 669 were imported. Almost all of the imported cases came from various neighboring Myanmar cities, particularly Mujie. The following is a breakdown of these cases by year: 238 (145 indigenous and 93 imported) in 2013; 297 (139 indigenous and 158 imported) in 2014; and 710 (309 indigenous and 418 imported) in 2015.

In 2015, emerging indigenous DF was reported in Gengma County in Lincang Prefecture. This outbreak included 96 indigenous and 104 imported cases. Imported patients originated from the neighboring Myanmar cities of Gungnong and Lashio.

#### Seasonal distribution of dengue cases

**Fig. 2:**
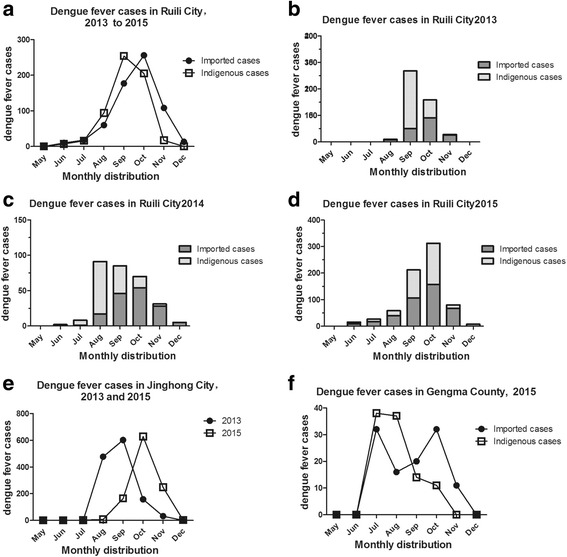
Monthly distribution of dengue fever cases in Yunnan Province, China, 2013–2015. **a** Monthly distribution of all indigenous and imported dengue fever cases in Ruili City, 2013–2015. **b**–**d**. Monthly distribution of indigenous and imported dengue fever cases in Ruili City in 2013 **b**, 2014 **c**, and 2015 **d**. **e** Monthly distribution of indigenous dengue fever cases in Jinghong City, 2013 and 2015. *There were more indigenous cases than imported cases in Jinghong, thus analysis of monthly distribution only included indigenous dengue fever cases. **f** Monthly distribution of indigenous dengue fever cases in Gengma County, 2015

The monthly distribution of reported dengue cases is shown in Fig. 2a–f, which includes indigenous and imported cases. Distinct and regular seasonal features in the fluctuations of both types of cases were seen during epidemic years. Sporadic imported dengue cases were observed and reported during January–May, a slight increase was seen in June, cases generally peaked in September or October (depending on the year), and then cases gradually subsided in November (Fig. 2b–d).

In Ruili City, indigenous cases peaked in September, while imported cases peaked in October (Fig. 2a). June to December was an epidemic period for indigenous cases in Ruili City, Jinghong City, and Gengma County, with a peak occurring in July, August, September, or October according to the city and year. In Ruili City, the epidemic period for indigenous cases began earlier and extended longer with each passing year: August–November (2013), July–November (2014), and June–December (2015) (Fig. 2b–d). In Jinghong City, the epidemic periods for indigenous cases were August–November (2013) and July–December (2015) (Fig. 2e). The epidemic period for indigenous cases in Gengma County was July–November (2015) (Fig. 2f) (Additional file 1).

#### Demographic distribution

**Fig. 3:**
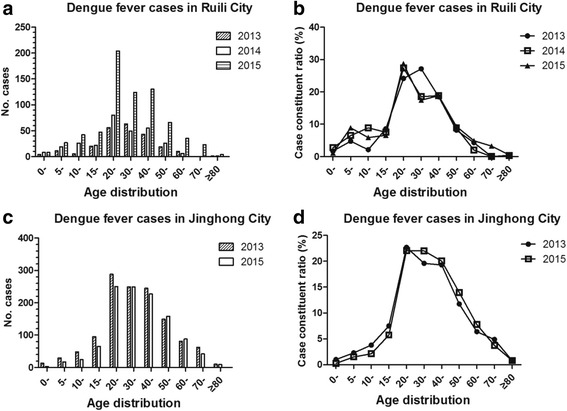
Age distribution of dengue fever cases in Yunnan Province of China, 2013–2015. Age distribution **a** and case constituent ratio **b** of age composition of dengue fever cases in Ruili City, 2013–2015. Age distribution **c** and case constituent ratio **d** of age composition of dengue fever cases in Jinghong City, 2013–2015

Of the 3634 DF cases from the cities of Ruili and Jinghong, 49.26% (1790) were male patients and 50.74% (1844) were female patients. Approximately 66.40% of the patients were adults (20–49 years of age) (Fig. 3b and d).

In Ruili City, 53.37% (661) of the DF cases were male patients and 46.43% (573) were female patients. The youngest patient was 6 months of age and the oldest was 86 years of age; 804 of these patients were 20–49 years of age, which accounted for 65.15% of all Ruili City DF cases. In 2013, a main peak appeared in the 30–39-year-old group; in 2014 and 2015, a main peak appeared in the 20–29-year-old group, while a secondary peak appeared in the 40–49-year-old group (Fig. 3a and b). The results of one-way ANOVA showed that there were significant differences (*P* < 0.001) according to age; the case constituent ratio (%) of the 20–49-year-old groups (20-, 30-, and 40-year-old groups) was significantly different among groups by Tukey’s multiple comparison test (*P* < 0.001). There were no observed differences in age (*P* > 0.05) between 20- to 30- and 30- to 40-year-old groups, and no observed differences in the 0–19-year-old groups (0- vs 5- vs 10- vs 15-year-old groups, *P* > 0.05).

In Jinghong City, 52.96% (1271) of the DF cases were female patients and 47.04% (1129) were male patients. The youngest patient was 5 months of age and the oldest patient was 89 years of age; approximately 67.04% of these patients were in the young adults group (20–49 years of age) (Fig. 3c and d). There were significant differences according to age (one-way ANOVA, *P* < 0.001); the case constituent ratio (%) of the 20–49-year-old groups (20-, 30-, and 40-year-old groups) was significantly different by Tukey’s multiple comparison test (*P* < 0.001). There were no observed differences in age (*P* > 0.05) between 20-, 30-, and 40-year-old groups, and no observed differences in the 0–19-year-old groups (0- vs 5- vs 10- vs 15-year-old groups, *P* > 0.05).

#### Occupational distribution of DF patients

Of the 3634 DF cases from the cities of Ruili and Jinghong, the most common occupational group was business and service personnel (30.82%). The next most common group of patients was housekeepers, unemployed, and retired personnel (22.07%), followed by peasants and peasant workers (11.70%) and school-age children (9.41%).

#### Dengue diagnosis, virus identification, and amplification of *CprM* and *E* genes

A total of 165 samples were available to amplify and sequence the *CprM* gene from 232 DENV NS1 antigen-positive sera, which were randomly collected from DF patients in Yunnan Province, 2013–2015 (Table 2). Among 90 randomly-selected acute-phase sera (within 5 days of disease onset) that were positive for DENV NS1 antigen and used for *CprM* gene amplification, 88 DENV isolates were successfully obtained after inoculation and culture in C6/36 cells. These isolates were reverse transcribed by RT-PCR, followed by sequencing of *CprM* (Additional file 2) and *E* genes, and the sequences were submitted to the NCBI GenBank database (GenBank accession numbers: KX262914–KX262958 and KX056445–KX056474).

**Table 2:** Serotype Identification and Amplification of the *CprM* Gene of dengue fever cases in Yunnan Province, China, 2013 to 2015

Prefecture	2013		2014		2015		Total
	Indigenous	Imported	Indigenous	Imported	Indigenous	Imported	
Kunming		***2/4*** ^a^					***2/4***
		I (2)^b^					I (2)
Lincang					***5/8***	***8/10***	***13/18***
					I (5)	I (8)	I (13)
Dehong	***7/10***	***2/5***	***22/37***	***27/42***	***35/38***	***26/33***	***119/165***
	I (2)	I (1)	I (19)	I (26)	I (16)	I (10)	I (74)
	II (5)	II (1)	II (3)	II (1)	II (17)	II (11)	II (38)
					IV (2)	IV (5)	IV (7)
Xishangbanna	***11/12***	***1/3***			***17/25***	***2/5***	***31/45***
	III (11)	III (1)			II (17)	II (2)	II (19) III (12)

^a^The denominator was the number of the positive acute serum of DENV NS1 antigen to Amplification of the *CprM,* and the numerator was the number of positive acute serum of Amplificating the DENV *CprM* in the test samples. ^b^: I, II, III and IV is the 4 distinct serotypes: DENV-1, DENV-2, DENV-3 and DENV-4, respectively; the number in the bracket is the number of DENV serotype

#### Phylogenetic analysis of isolated DENV and homologous strains reported in Yunnan and Guangdong Provinces and Southeast Asian countries

Phylogenetic analysis was performed based on the sequences obtained, including reference sequences from the NCBI GenBank database. This analysis indicated that DENV strains of all four serotypes were present in indigenous cases in Yunnan Province: DENV-1, DENV-2, and DENV-4 in Ruili City from 2013 to 2015; DENV-2 and DENV-3 in Xishuangbanna Prefecture in 2013 and 2015; and DENV-1 in Gengma County in 2015 (Table 2).

**Fig. 4:**
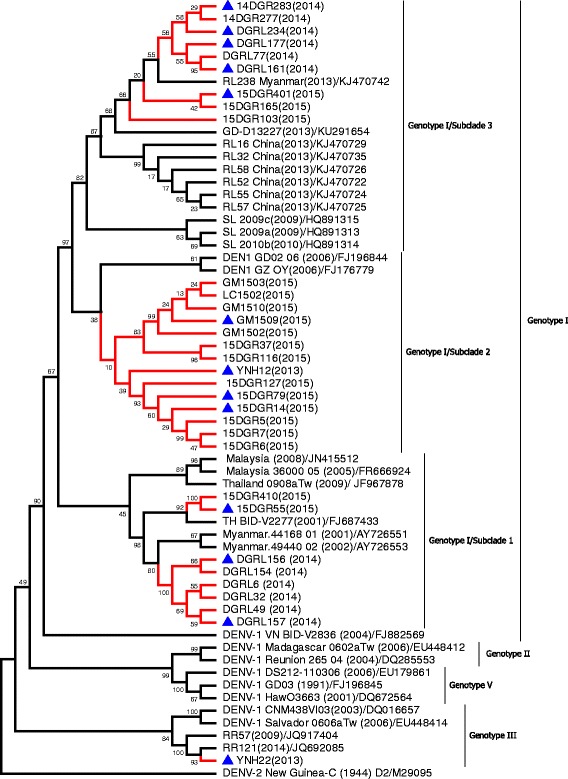
Phylogenetic analysis of isolated DENV-1 *E* gene sequences with homologous strains reported in Yunnan Province. The phylogenetic tree was generated in MEGA version 6 (www.megasoftware.net) using the neighbor-joining method with 1000 bootstrap replicates. Viruses isolated from indigenous and imported cases evaluated in this paper are highlighted using red branches (isolates from imported cases are labeled with *blue* triangles). The isolates RL16–RL58, DGRL6–DGRL283, and 15DGR5–15DGR410 were isolated from Ruili City in 2013, 2014, and 2015, respectively. The isolates GM1502–GM1510 and LC1502 were isolated from Gengma County and Linxiang County of Lincang Prefecture in 2015. The isolates YNH22 andYNH12 were isolated from Kunming Prefecture in 2013

Homological analysis showed that DENV-1 found in Ruili City (2013–2015) and Gengma County (2015) was genotype I, which was the predominant genotype circulating in Southeast Asian countries. The phylogenetic tree revealed that DENV-1 genotype I showed 3 subclades and was named provisional genotype I/subclades 1–3 in DENV-1 (Fig. 4). The isolates of provisional genotype I/subclade 1 circulating in 2014 and 2015 were closely related to viruses from Thailand and Myanmar. The isolates of subclade 2 circulating in Ruili City and Gengma County (2015) clustered with viruses from Guangdong Province (2006). The isolates of subclade 3, which were circulating in Ruili City (2013–2015), were closely related to viruses from Guangdong Province and Myanmar and clustered with viruses from Sri Lanka (2009).

**Fig. 5:**
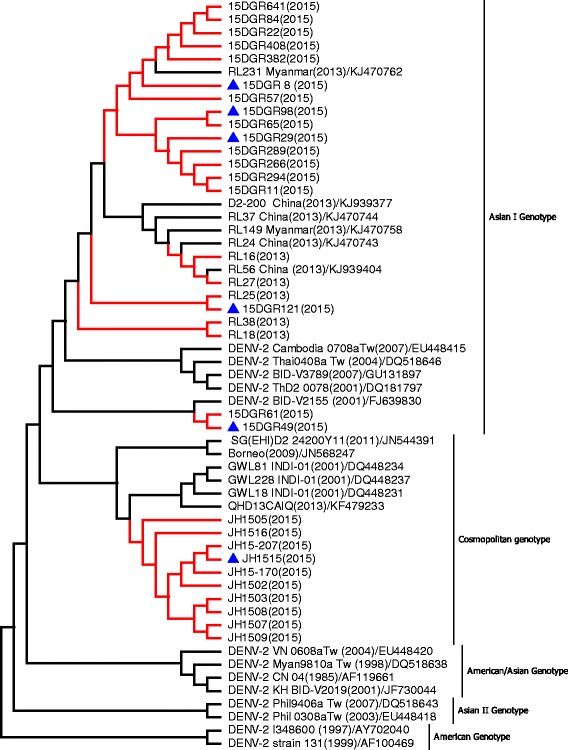
Phylogenetic analysis of isolated DENV-2 *E* gene sequences with homologous strains reported in Yunnan Province. The phylogenetic tree was generated in MEGA version 6 using the neighbor-joining method with 1000 bootstrap replicates. Viruses isolated from indigenous and imported cases evaluated in this paper are highlighted using red branches (isolates from imported cases are labeled with *blue* triangles). The isolates RL16–RL54 and 15DGR8–15DGR641 were isolated from Ruili City of Dehong Prefecture in 2013 and 2015, respectively. The isolates JH1502–JH15–207 were isolated from Jinghong City of Xishuangbanna Prefecture in 2015

Phylogenetic analysis indicated DENV-2 from Yunnan Province had a different genotype. The isolates from Ruili City during 2013–2015 were categorized into 5 subclades, which were classified as DENV-2 Asian I genotype, and clustered with viruses from Thailand and Myanmar. The isolates from Jinghong City (2015) formed a tight cluster and were identified as DENV-2 Cosmopolitan genotype (or genotype IV), which is closely related to viruses from India and China (Fig. 5).

**Fig. 6:**
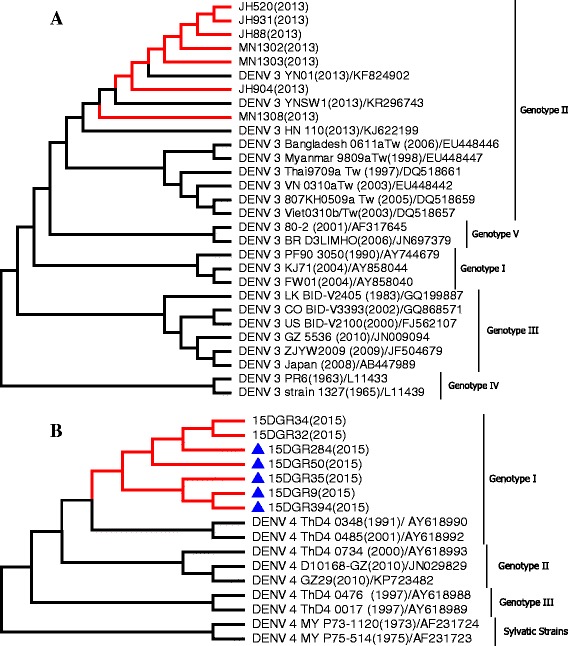
Phylogenetic analysis of isolated DENV *E* gene sequences with homologous strains reported in Yunnan Province. The phylogenetic tree was generated in MEGA version 6 using the neighbor-joining method with 1000 bootstrap replicates. Viruses isolated from indigenous and imported cases evaluated in this paper are highlighted using red branches (isolates from imported cases are labeled with *blue* triangles). The isolates JH88–JH931 and MN1302–MN1308 were isolated from Jinghong City and Mengla County of Xishuangbanna Prefecture in 2013, respectively; the isolates 15DGR9–15DGR394 were isolated from Ruili City of Dehong Prefecture in 2015. **a** DENV-3, **b** DENV-4

All isolates from Jinghong City and Mengla County in Xishuangbanna Prefecture in 2013 formed a tight cluster in the phylogenetic tree (Fig. 6) and were classified as DENV-3 genotype II. These isolates were closely related to viruses from Thailand, Myanmar, Bangladesh, Vietnam, and China. The seven isolates from Ruili City in 2015 were identified as DENV-4 genotype I (Fig. 6) and clustered with viruses from Thailand (1991).

## Discussion

Since 2000, sporadic imported cases of DF have been reported almost annually in Yunnan Province [24]. In 2008, an epidemic of DF imported from Mujie City, Myanmar was reported in Ruili City, Yunnan Province [25]. In 2008, a smaller epidemic of 12 cases of indigenous DF occurred in Nansan (Zhengkang County) and Manghai (Mangshi County), which are located in southwestern Yunnan Province and border Myanmar, the suspected source of imported DF cases. In the current study, we confirmed that a large-scale indigenous DF outbreak emerged during 2013–2015 in Jinghong City, Ruili City, and Gengma County, each of which share borders with Laos and Myanmar. In Jinghong City, Xishuangbanna Prefecture, indigenous DF outbreaks of DENV-3 (2013) and DENV-2 (2015) occurred. DENV-1 and DENV-2 emerged annually in Ruili City during 2013–2015, and DENV-4 emerged in 2015. An indigenous DF outbreak of DENV-1 also emerged in Gengma County in 2015.

All dengue infection cases reported in Yunnan Province during 2013–2015 were DF, and not dengue hemorrhagic fever (DHF). In Thailand, DHF was first recognized in 1949 [30]. Annually, DF/DHF cases are mostly observed in the 5–9-year-old group and the number of cases in the 5–14-year-old group accounts for 70–75% of all reported cases in Thailand [30, 31]. With respect to the age distribution of dengue cases in Yunnan Province, a comparatively higher incidence (66.40% of all DF patients) of infection was observed in the 20–49-year-old group. In the 0–14-year-old group, the number of cases accounted for 3.89–7.08% of all reported cases in Jinghong City and 8.62–18.15% in Ruili City. This pattern differed from that observed in many dengue-endemic areas in Southeast Asian countries such as Thailand, in which children were most commonly infected [2, 32]. These results indicated that epidemic dengue in Yunnan Province has emerged in new geographic regions. In 2015, epidemic transmission of dengue in Gengma County occurred in rural villages. However, in Jinghong City and Ruili City, epidemic transmission occurred in large, tropical, urban centers and was part of the urban endemic/epidemic cycle, which is the most important transmission cycle from a public health standpoint [32].

As four major drivers, failure to control *Ae. aegypti* mosquitoes in urban environments, lifestyle changes, urbanization, and globalization have increased the incidence and geographic spread of epidemic dengue [6]. Yunnan Province contains both tropical and subtropical regions with a 4060-km border with Southeast Asian countries. Previous investigations have confirmed that *Ae. albopictus* mosquitoes are widely distributed in the province [33, 34]; before 2004, however, *Ae. aegypti* mosquitoes were not found in this region. In 2004, *Ae. aegypti* mosquitoes were found only in the Jiegao Port area of Ruili City; by 2009, investigations of mosquito vectors again confirmed that *Ae. aegypti* mosquitoes were distributed locally around Jinghong Port (a port on the Lancang River, also known as the Mekong River) [35]. These results revealed that *Ae. aegypti* has expanded geographically from Southeast Asian countries to ports along the China-Myanmar and China-Laos borders through transport and cargo vessels.

In recent years, population densities and geographic distribution of *Ae. aegypti* have increased, especially in urban areas of Yunnan Province [35]. In 2013, investigations of mosquito vectors by the Xishuangbanna CDC and Ruili CDC revealed that the population densities of *Ae. aegypti* are greater than those of *Ae. albopictus*. The constituent ratios of *Ae. aegypti* and *Ae. albopictus* in Ruili City were 82.06% and 18.94%, respectively; in Jinghong City, they were 59.89% and 40.11%, respectively. Comprehensive analysis indicated that the indigenous dengue outbreak in Yunnan Province may be closely related to the expansion and invasion of *Ae. aegypti* in this region. Both *Ae. aegypti* and *Ae. albopictus* can transmit DENV to humans, and their presence in urban areas of Yunnan Province increases the risk of indigenous transmission. In Ruili City, the epidemic period for indigenous cases began earlier and extended longer with each passing year from 2013 to 2015. This may be affected or correlated with meteorological factors such as warming and precipitation enhancement in the rainy season, which has increased mosquito population densities and resulted in failure to control mosquitoes.

The two most common occupational groups identified among DF patients were business and service personnel (30.82%) and housekeepers, unemployed, and retired personnel (22.07%). It is important to note that residents work and live in environments that are densely populated with *Ae. aegypti* during the day, which increases exposure to infectious mosquito bites and DENV.

The emergence of DHF is associated with introduction of multiple strains of each serotype and the development of hyperendemicity in the region [6, 36]. Phylogenetic analysis indicated that DENV strains of all four serotypes have been identified in indigenous cases in Yunnan Province. During 2013–2015, DENV-1 genotype I, DENV-2 Asian I genotype, and DENV-4 genotype I were identified in Ruili City. The dengue epidemic pattern observed in Ruili City demonstrated hypoendemic characteristics: circulation of DENV-1 and DENV-2 over consecutive years. Multiple strains of each serotype were introduced, which may increase the risks of DHF emergence in Ruili City. All isolates from Xishuangbanna Prefecture in 2013 were classified as DENV-3 genotype II and isolates in 2015 were classified as DENV-2 Cosmopolitan genotype (or genotype IV). Fortunately, DENV-3 genotype II and DENV-2 Asian I genotype were not detected in dengue cases from Xishuangbanna Prefecture in 2015.

## Conclusions

The present study suggested that multiple DENV serotypes are endemic in countries bordering Yunnan Province such as Laos, Thailand, and Myanmar. Imported DF patients from Laos and Myanmar were the primary cause of DF epidemics in Yunnan Province, and *Ae. aegypti* and *Ae. albopictus* remain widely distributed in urban regions of the tropics. These factors increase the risk of indigenous dengue transmission and indigenous DF epidemics or pandemics in Yunnan Province. Such evidence indicated that Ruili City is increasingly exhibiting features of an endemic area, as suggested by sustained DENV and the co-existence of multiple serotypes in this area. To prevent future dengue outbreaks and the spread of disease to other regions, joint dengue prevention and control measures should be strengthened in the China-Myanmar and China-Laos border regions of Yunnan Province. In particular, a sound laboratory-based disease surveillance system of imported patients and integrated vector control management are required in both of these regions.

## Additional files

Additional file 1: Dengue fever cases 2013–2015 in YunnanR3. (XLSX 11 kb)

Additional file 2: Figure S1. Figure Appendix. Phylogenetic analysis of isolated DENV-1, 2, 3 and 4 *CprM* gene sequences along with the homologous strains reported in Yunnan Province and related reference viruses retrieved from the GenBank database. (PDF 73 kb)

## Abbreviations

CDC: Center for Disease Control and Prevention
CPE: Cytopathic effects
CprM: Capsid/premembrane region
DENV: Dengue virus
DF: Dengue fever
DHF: Dengue hemorrhagic fever
E: Envelope

## References

[1] Kraemer MU, Sinka ME, Duda KA, Mylne AQ, Shearer FM, Barker CM, Moore CG, Carvalho RG, Coelho GE, Van Bortel W (2015). The global distribution of the arbovirus vectors *Aedes aegypti* and Ae albopictus. elife.

[2] WHO: Geneva. Dengue: Guidelines for Diagnosis, Treatment, Prevention and Control: New Edition. WHO and the Special Programme for Research and Training in Tropical Diseases 2009:3–16.

[3] Wang E, Ni H, Xu R, Barrett AD, Watowich SJ, Gubler DJ, Weaver SC (2000). Evolutionary relationships of endemic/epidemic and sylvatic dengue viruses. J Virol.

[4] Bhatt S, Gething PW, Brady OJ, Messina JP, Farlow AW, Moyes CL, Drake JM, Brownstein JS, Hoen AG, Sankoh O (2013). The global distribution and burden of dengue. Nature.

[5] Massad E, Coutinho FA (2011). The cost of dengue control. Lancet.

[6] Gubler DJ (2011). Dengue, Urbanization and Globalization: The Unholy Trinity of the 21(st) Century. Trop Med Health.

[7] Wilder-Smith A, Gubler DJ (2008). Geographic expansion of dengue: the impact of international travel. Med Clin N Amer.

[8] Wilder-Smith A, Schwartz E (2005). Dengue in travelers. N Engl J Med.

[9] Gubler DJ (1998). The global pandemic of dengue/dengue haemorrhagic fever: current status and prospects for the future. Ann Acad Med Singap.

[10] Holmes EC, Tio PH, Perera D, Muhi J, Cardosa J (2009). Importation and co-circulation of multiple serotypes of dengue virus in Sarawak, Malaysia. Virus Res.

[11] Jarman RG, Holmes EC, Rodpradit P, Klungthong C, Gibbons RV, Nisalak A, Rothman AL, Libraty DH, Ennis FA, Mammen MP (2008). Microevolution of Dengue viruses circulating among primary school children in Kamphaeng Phet, Thailand. J Virol.

[12] Malla S, Thakur GD, Shrestha SK, Banjeree MK, Thapa LB, Gongal G, Ghimire P, Upadhyay BP, Gautam P, Khanal S (2008). Identification of all dengue serotypes in Nepal. Emerg Infect Dis.

[13] Thant KZ, Tun MM, Parquet Mdel C, Inoue S, Lwin YY, Lin S, Aye KT, Khin PT, Myint T, Htwe K (2015). Molecular Epidemiology of Dengue Viruses Co-circulating in Upper Myanmar in 2006. Trop Med Health.

[14] Trent DW, Grant JA, Monath TP, Manske CL, Corina M, Fox GE (1989). Genetic variation and microevolution of dengue 2 virus in Southeast Asia. Virology.

[15] Nguyen NH, Tran VB, Morris GE (1997). Nucleotide sequences from the capsid and pre-protein regions of dengue viruses from Vietnam. Biochem Soc Trans.

[16] Russell PK, Van Quy D, Nisalak A, Simasathien P, Yuill TM, Gould DJ (1969). Mosquito vectors of dengue viruses in South Vietnam. AmJTrop Med Hyg.

[17] Dubot-Peres A, Vongphrachanh P, Denny J, Phetsouvanh R, Linthavong S, Sengkeopraseuth B, Khasing A, Xaythideth V, Moore CE, Vongsouvath M (2013). An epidemic of dengue-1 in a remote village in rural Laos. PLoS Negl Trop Dis.

[18] Vu TT, Holmes EC, Duong V, Nguyen TQ, Tran TH, Quail M, Churcher C, Parkhill J, Cardosa J, Farrar J (2010). Emergence of the Asian 1 genotype of dengue virus serotype 2 in viet nam: in vivo fitness advantage and lineage replacement in South-East Asia. PLoS Negl Trop Dis.

[19] Jing QL, Yang ZC, Luo L, Xiao XC, Di B, He P, Fu CX, Wang M, Lu JH (2012). Emergence of dengue virus 4 genotype II in Guangzhou, China, 2010: survey and molecular epidemiology of one community outbreak. BMC Infect Dis.

[20] Luo L, Liang HY, Hu YS, Liu WJ, Wang YL, Jing QL, Zheng XL, Yang ZC (2012). Epidemiological, virological, and entomological characteristics of dengue from 1978 to 2009 in Guangzhou, China. J Vector Ecol.

[21] Shen SQ, Wei HX, Fu YH, Zhang H, Mo QY, Wang XJ, Deng SQ, Zhao W, Liu Y, Feng XS (2015). Multiple Sources of Infection and Potential Endemic Characteristics of the Large Outbreak of Dengue in Guangdong in 2014. Sci Rep.

[22] Wang B, Li Y, Feng Y, Zhou H, Liang Y, Dai J, Qin W, Hu Y, Wang Y, Zhang L (2015). Phylogenetic analysis of dengue virus reveals the high relatedness between imported and local strains during the 2013 dengue outbreak in Yunnan, China: a retrospective analysis. BMC Infect Dis.

[23] Wang B, Yang H, Feng Y, Zhou H, Dai J, Hu Y, Zhang L, Wang Y, Baloch Z, Xia X (2016). The distinct distribution and phylogenetic characteristics of dengue virus serotypes/genotypes during the 2013 outbreak in Yunnan, China: Phylogenetic characteristics of 2013 dengue outbreak in Yunnan, China. Infect Genet Evol.

[24] Wu JY, Lun ZR, James AA, Chen XG (2010). Dengue Fever in mainland China. AmJTrop Med Hyg.

[25] Zhang HL, Fu SH, Deng Z, Yuan J, Jiang HY, Li MH, Gao XY, Wang JL, Liu YH, Yin ZL (2013). An outbreak of imported dengue fever from Myanmar to the border of China, with its viral molecular epidemiological features. Zhonghua liu xing bing xue za zhi.

[26] Guo X, Yang H, Wu C, Jiang J, Fan J, Li H, Zhu J, Yang Z, Li Y, Zhou H (2015). Molecular Characterization and Viral Origin of the First Dengue Outbreak in Xishuangbanna, Yunnan Province, China, 2013. AmJTrop Med Hyg.

[27] Lanciotti RS, Calisher CH, Gubler DJ, Chang GJ, Vorndam AV (1992). Rapid detection and typing of dengue viruses from clinical samples by using reverse transcriptase-polymerase chain reaction. J Clin Microbiol.

[28] Tamura K, Stecher G, Peterson D, Filipski A, Kumar S (2013). MEGA6: Molecular Evolutionary Genetics Analysis version 6.0. Mol Biol Evol.

[29] Chew MH, Rahman MM, Hussin S (2015). Molecular epidemiology and phylogenetic analysis of Dengue virus type-1 and 2 isolated in Malaysia. Pak J Med Sci.

[30] Yongyuth W (1997). Dengue Control through Schoolchildren in Thailand. Dengue Bull.

[31] WHO and the Special Programme for Research and Training in Tropical Diseases. Dengue guidelines for diagnosis, treatment, prevention and control: new edition[R]. Geneva: WHO; 2009. pp. 3-16.

[32] Gubler DJ (1998). Dengue and dengue hemorrhagic fever. Clin Microbiol Rev.

[33] Wang Piyu WC, Canglin Z (2006). Survey of transmission vectors of dengue fever in some areas in Yunnan Province, China. Trop Med.

[34] Zhang Hailin ZD, Zhuqing M (2001). Characterized distribution of *Aedes albopictus* and their relation with arbovirus in Yunnan Province. Chin J Vector Biol Control.

[35] Yang Mingdong JJ, Yu-ting Z, Hongning Z (2015). Distribution survey on *Aedes aegypti* in the border areas of Yunnan province, China. Chin J Vector Biol Control.

[36] Gubler DJ, Trent DW (1993). Emergence of epidemic dengue/dengue hemorrhagic fever as a public health problem in the Americas. Infect Agents Dis.

