# Supplementary material for: Epidemiological and molecular characteristics of emergent dengue virus in Yunnan Province near the China-Myanmar-Laos border, 2013–2015
Source: BMC Infect Dis. 2017 May 8;17:331. doi: 10.1186/s12879-017-2401-1 (PMC5422898; doi:10.1186/s12879-017-2401-1)
Supplement: Supplementary file 2 — Figure Appendix. Phylogenetic analysis of isolated DENV-1, 2, 3 and 4 CprM gene sequences along with the homologous strains reported in Yunnan Province and related reference viruses retrieved from the GenBank database. (PDF 73 kb) [file 12879_2017_2401_MOESM2_ESM.pdf]

Figure Appendix

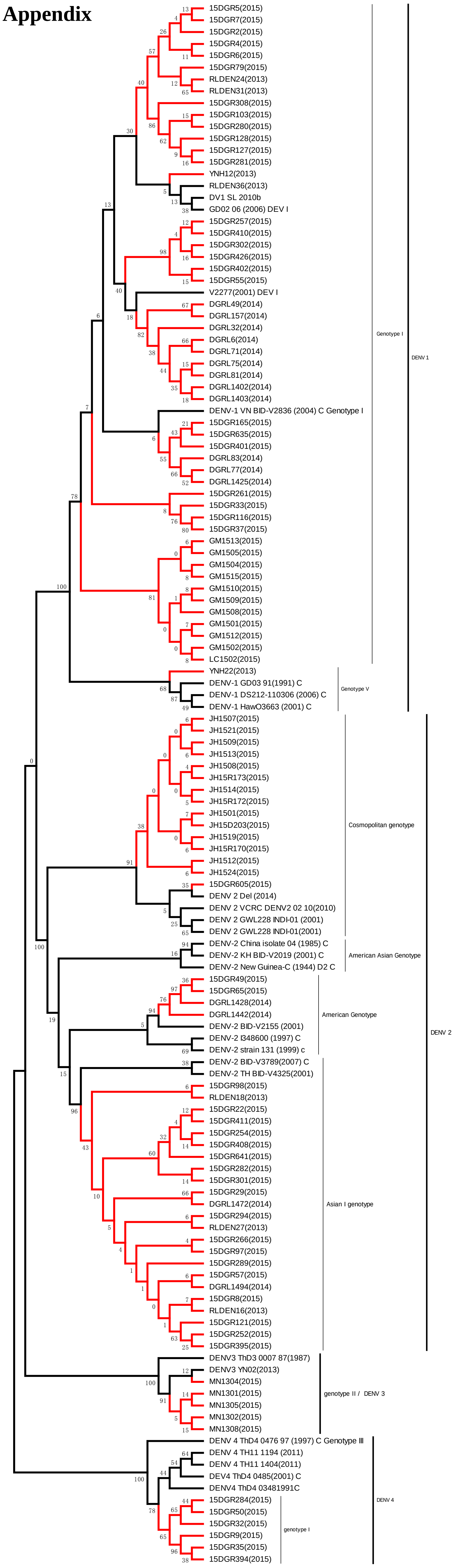

Figure Appendix. Phylogenetic analysis of isolated DENV-1, 2, 3 and 4 *CprM* gene sequences along with the homologous strains reported in Yunnan Province and related reference viruses retrieved from the GenBank database.

The phylogenetic tree was generated in MEGA version 6 (www.megasoftware. net), using Neighbor-joining analysis with 1,000 bootstrap replicates. The viruses isolated from indigenous and imported cases in this paper were highlighted using red branchs (and imported cases isolates labeled with blue triangles). The isolates RLDEN16 to RLDEN31, DGRL6 to DGRL1494 and 15DGR5 to 15DGR641 were isolated from Ruili city in 2013, 2014 and 2015, respectively. The isolates GM1501 to GM1515 and LC1502 were isolated from Gengma and Linxiang county of Lincang Prefecture in 2015; the isolates YNH22 andYNH12 were isolated from Kunming in 2013. The isolates JH1501 to JH15D207 were isolated from Jinghong city of Xishuangbanna Prefecture in 2015. The isolates MN1301 to MN1308 were isolated from Mengla city of Xishuangbanna Prefecture in 2013.
